# Complete Genome Sequence of European Genotype Porcine Reproductive and Respiratory Syndrome Virus Strain LNEU12 in Northern China

**DOI:** 10.1128/genomeA.00957-14

**Published:** 2014-10-09

**Authors:** Bing Li, Shenyang Gao, Tiezhong Zhou, Xiaogang Liu, He Lu, Fangzhou Feng

**Affiliations:** a College of Animal Husbandry and Veterinary Medicine, Liaoning Medical University, Jinzhou, Liaoning, People’s Republic of China; b College of Veterinary Medicine, Jilin University, Changchun, Jilin, People’s Republic of China

## Abstract

We report the complete genome sequence of a European genotype porcine reproductive and respiratory syndrome virus isolated from swine in northern China in 2012. Genome alignment revealed that the virus (LNEU12) strain shares 90.1% nucleotide identity with the European prototype Lelystad virus. Here, we announce the complete genome sequence of LNEU12.

## GENOME ANNOUNCEMENT

The porcine reproductive and respiratory syndrome virus (PRRSV) is an enveloped RNA virus, belonging to the *Arteriviridae* family, that is a significant cause of swine morbidity and mortality worldwide (1, 2). PRRSV is endemic in most swine-producing countries and causes enormous economic losses in the swine industry (3). Currently, two main genotypes of PRRSV are recognized, European (genotype 1) and North American (genotype 2), which have only about 60% nucleotide identity at the genome level (4). Since PRRSVs were first reported in 1996, all heretofore described PRRSVs in China, including the HP-PRRSV isolates, have belonged to the North American genotype (5, 6, 7, 8). However, a few partial European PRRSVs were isolated in mainland China recently, such as BJEU06-1, NMEU09-1, and NVDC-NM1-2011 (9, 10). To further explore the molecular epidemiological characteristics of the European genotype of PRRSV in China, we report here the complete genome sequence of LNEU12.

The LNEU12 PRRSV was isolated from serum samples of piglets suspected to be infected in northern China in 2012. The complete genome sequence was generated with PCR using 15 pairs of oligonucleotide primers to amplify different regions of the PRRSV. The PCR products were purified and cloned into pMD18-T vector (TaKaRa), sequenced with an ABI3730xl genome sequencer, and assembled using DNAStar version 7.1 to obtain the complete genome sequence. The whole genome of LNEU12 is 15,083 bp, not including poly(A) sequences. Genetic analyses demonstrated that LNEU12 exhibits 90.1%, 93.1%, and 90.8% sequence identities with PRRSV strains Lelystad virus (LV), NVDC-NM1-2011, and BJEU06-1, respectively, and 57.5% sequence identity with the North American prototype VR2332, indicating that it belongs to EU-PRRSV.

However, LNEU12 has 18-nt discontinuous deletions in comparison with the EU-PRRSV prototypic of strain Lelystad virus by DNAStar version 7.1, which are distributed in different regions as follows: 12 nt(ATTAACCTGGTA) deletions at positions 2,448 to 2,459, 0.3 nt(GTG) deletions at positions 2,608 to 2,610, and 3 nt(CCT) deletions at positions 13,142 to 13,144. Moreover, LNEU12 has similar characteristics to BJEU06-1, NMEU09-1, and NVDC-NM1-2011 (9, 10) at the deletions sites, which indicates that they probably evolved from similar progenitors. Our study indicates that the EU genotype PRRSV variant continues to have a prevailing and accelerating evolution in China. The genome data of LNEU12 will help us to elucidate the molecular characteristics and evolution trend of PRRSV.

### Nucleotide sequence accession number.

The complete genome sequence of LNEU12 is available in GenBank under the accession number KM196101.
